# Retroperitoneal glass foreign body with renal parenchymal involvement after 25 years of silent retention: a case report

**DOI:** 10.3389/fsurg.2026.1878687

**Published:** 2026-08-25

**Authors:** Gagik N. Akopyan, Mikhail D. Mutsenieks, Suleiman A. Yandiev, Elizaveta I. Timashova, Denis V. Chinenov, Evgeny V. Shpot, Petr V. Glybochko

**Affiliations:** Institute for Urology and Human Reproductive Health, Sechenov First Moscow State Medical University, Moscow, Russia

**Keywords:** 3D reconstruction, CT imaging, delayed presentation, glass fragment, gross haematuria, laparoscopy, renal trauma, retroperitoneal foreign body

## Abstract

We report a 46-year-old woman in whom a glass foreign body retained in the retroperitoneum for 25 years caused recurrent exertional gross haematuria after apparent migration through the quadratus lumborum muscle into the upper renal pole and calyceal system. A penetrating thoracolumbar injury sustained in a motor vehicle accident 25 years earlier had gone untreated. Initial evaluation at an outside institution attributed the symptoms to nephrolithiasis for over 12 months. Multiphase contrast-enhanced CT demonstrated two hyperdense foreign-body fragments (∼1,150–1,200 HU) along a single track extending from the right L5 transverse process through the quadratus lumborum into the renal parenchyma, consistent with a fractured retained glass foreign body. Volumetric 3D CT reconstruction delineated the fragment trajectory in three planes and was consistent with the absence of major vascular encasement. Flexible ureterorenoscopy visualised glass within the renal pelvis, and endoscopic-only retrieval was considered unfeasible. Laparoscopic transperitoneal removal was performed: after medial mobilisation of the ascending colon and incision of Gerota's fascia, both fragments were dissected free under magnified direct vision and removed completely—a proximal fragment (∼3 cm) from the quadratus lumborum and a distal fragment (∼4 cm) from the renal parenchyma. No vascular or parenchymal injury occurred; the patient was discharged after an uneventful 7-day postoperative stay. To our knowledge, and based on the available published literature, no prior case describes a retroperitoneal glass foreign body with renal parenchymal involvement after a comparable 25-year retention interval. This case highlights three practical lessons: a systematic trauma history is valuable in unexplained haematuria; multiphase CT with 3D reconstruction can provide both diagnosis and a surgical roadmap; and laparoscopic transperitoneal removal was safe and effective in this patient once vascular involvement had been excluded preoperatively.

## 1 Introduction

Retained retroperitoneal foreign bodies are among the least common entities encountered in urology and general surgery (1, 2). Most reported cases are iatrogenic—broken instruments or retained surgical material—whereas foreign bodies of penetrating traumatic origin form a smaller and probably underreported subset (3, 4).

Because such foreign bodies may remain clinically silent, affected patients can present years or decades after the original injury with nonspecific symptoms—gross haematuria, renal colic, or flank pain—that are readily attributed to common conditions, most often nephrolithiasis (5).

The link to a remote penetrating injury is easily overlooked unless a directed trauma history is obtained; a systematic trauma inquiry is therefore an important step in the evaluation of otherwise unexplained haematuria (1, 5).

Glass in particular may be prone to prolonged retention. It is relatively chemically stable and is highly radiodense, so that it is often recognised on cross-sectional imaging only when a foreign body is specifically considered in the differential diagnosis (6, 7). Reported retention intervals for glass include more than 40 years in the maxillofacial region (8) and approximately 12–25 years in the soft tissues of the back (9).

Multiphase CT with volumetric 3D reconstruction is regarded as the standard for foreign-body localisation and surgical planning (6, 7, 10). Renal parenchymal involvement by a retained glass fragment after prolonged latency is, to our knowledge, rarely documented. In haemodynamically stable patients, laparoscopy is generally preferred over open exploration for retroperitoneal foreign-body retrieval, offering magnified visualisation and lower reported morbidity (2–4, 11). We present this case, together with a focused narrative review of comparable published reports, to describe the diagnostic pathway, the operative strategy, and the clinical lessons of relevance to practising urologists. This case report was prepared in accordance with the CARE guidelines (12, 13).

## 2 Case presentation

### 2.1 Clinical presentation

A 46-year-old woman (BMI 24 kg/m^2^) presented with a 12-month history of recurrent right flank pain and gross haematuria consistently precipitated by physical exertion (Table 1). All haematological, biochemical, and coagulation parameters were within normal limits. Urinalysis showed 12–15 erythrocytes per high-power field, including 3–4 dysmorphic forms.

**Table 1:** Chronological sequence of key clinical events.

Period	Clinical event
Dec 2024 – Jul 2025	Recurrent exertional gross haematuria and right renal colic. Outside institution: ultrasound misinterpreted as renal calculi; empirical antibiotic therapy initiated without clinical benefit.
Aug 2025	Admission to Rozanov City Clinical Hospital. CE-CT: linear high-density retroperitoneal structure misattributed to a drainage tube or calcification.
Oct 22, 2025	Repeat CE-CT: two hyperdense fragments of a fractured glass foreign body (∼1,150–1,200 HU) along a single track from the right L5 transverse process through quadratus lumborum into the upper renal pole and calyceal system. Volumetric 3D CT reconstruction and flexible ureterorenoscopy performed at Sechenov University Urology Clinic.
Nov 21, 2025	Elective laparoscopic transperitoneal removal under general anaesthesia. Complete removal of both glass fragments; no vascular or parenchymal injury. Discharged after a 7-day postoperative stay.

CE-CT, contrast-enhanced computed tomography.

### 2.2 Remote trauma history

Systematic history-taking revealed a penetrating right thoracolumbar injury sustained in a motor vehicle accident 25 years earlier, for which no medical care had been sought at the time.

### 2.3 Initial diagnosis and differential diagnosis

At the referring institution the symptoms had been attributed to intracalyceal calculi on ultrasound, and empirical antibiotic therapy had been initiated without clinical benefit. An initial contrast-enhanced CT (CE-CT, August 2025) attributed a linear high-density retroperitoneal structure to a drainage tube. The principal differential diagnosis was therefore nephrolithiasis; the absence of a convincing calculus, together with the eventual recognition of a remote penetrating injury, prompted reconsideration and dedicated re-imaging with a retained foreign body in the differential.

### 2.4 CT and ultrasound findings

**Figure 1:**
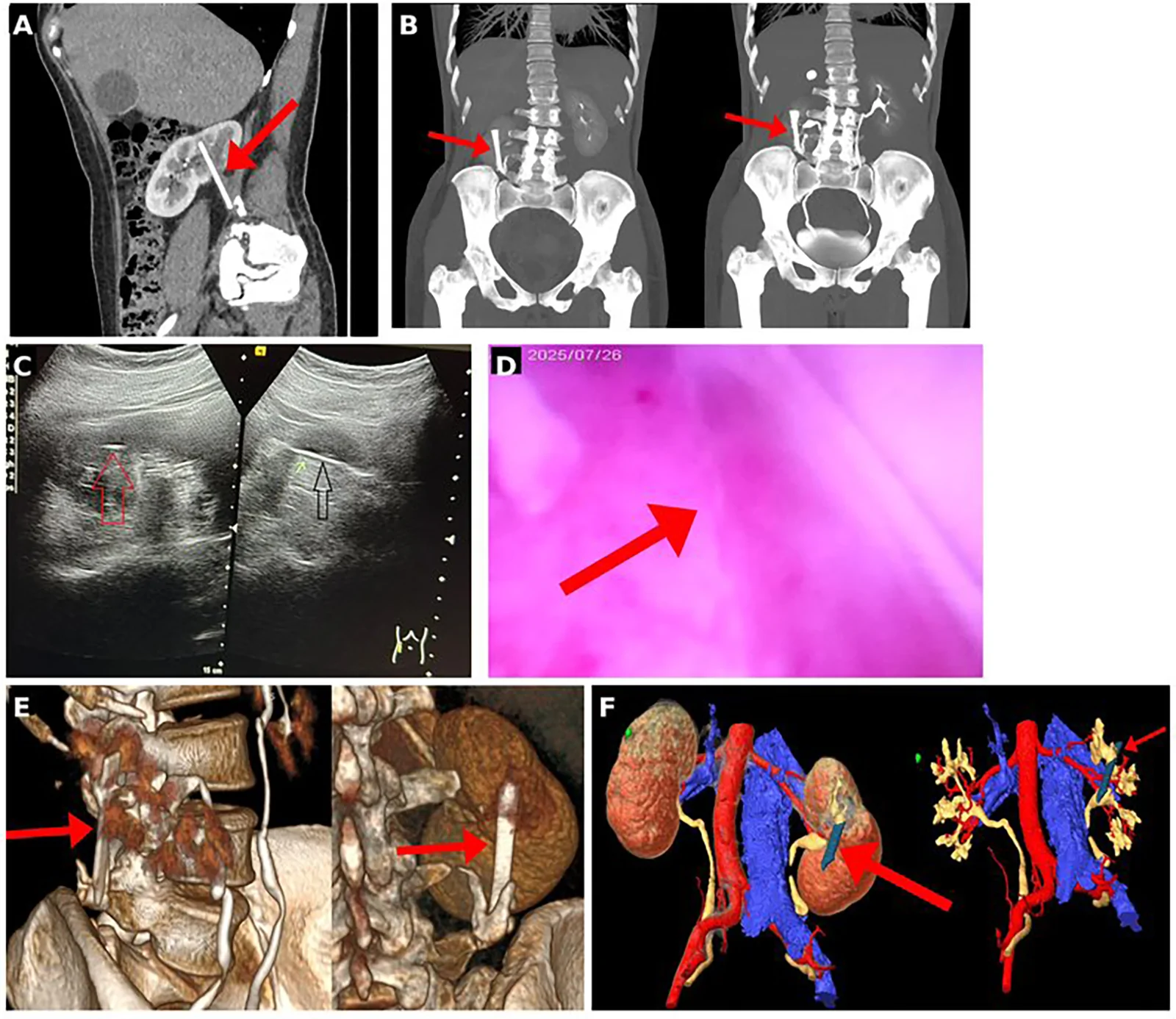
Preoperative multimodality imaging. **(A)** CE-CT sagittal reconstruction: hyperdense glass foreign-body material (arrow; ∼1,150–1,200 HU) extending from the right L5 transverse process through quadratus lumborum into the upper renal pole; the foreign body was present as two fragments along this track. **(B)** CE-CT coronal reconstruction, excretory phase: the foreign body (arrows) traverses renal parenchyma and enters the upper calyces, with no hydronephrosis or contrast extravasation; the proximal (quadratus lumborum) and distal (renal parenchyma) fragments correspond to the two components removed at surgery. **(C)** Transabdominal ultrasound, longitudinal view: linear hyperechoic foreign-body material (arrow) projecting beyond the renal capsule; colour Doppler confirms preserved segmental perfusion. **(D)** Flexible ureterorenoscopy: direct endoscopic visualisation of glass (arrow) within the renal pelvis. **(E,F)** Volumetric 3D CT reconstructions (posterior-oblique and anterior views): colour-coded segmentation shows the foreign body (teal) relative to the right kidney (orange), aorta (red), IVC (blue), and vertebral column (beige), delineating the oblique trajectory and supporting the absence of major vascular encasement.

Repeat multiphase CE-CT on 22 October 2025 (Figures 1A,B) revealed two adjacent hyperdense structures [∼1,150–1,200 HU, values consistent with glass (6, 7)] lying along a single track that extended from the right L5 transverse process through the quadratus lumborum into the upper renal pole, traversing renal parenchyma and the upper calyceal system. The distal structure lay within the renal parenchyma and upper calyx, and the more proximal structure lay within the quadratus lumborum and adjacent retroperitoneal fat; together they were interpreted as two fragments of a single fractured retained glass foreign body, an interpretation subsequently confirmed at operation. The right kidney measured 105 mm in longitudinal length with preserved corticomedullary differentiation, no cortical thinning, and no hydronephrosis; the left kidney measured 110 mm with preserved corticomedullary differentiation and no hydronephrosis. Neither haematoma nor urinary extravasation was present, and both collecting systems were unobstructed. Multiphase contrast analysis demonstrated no direct encasement of major vessels by either fragment.

Transabdominal ultrasound (Figure 1C) confirmed linear hyperechoic foreign-body material projecting beyond the renal capsule along the track, with preserved segmental perfusion on colour Doppler. Despite traversal of the upper calyceal system, no hydronephrosis was present: the foreign body lay eccentrically within the upper pole and calyx and did not obstruct the ureteropelvic junction or ureter, so that the pelvicalyceal system remained unobstructed on CT and at subsequent ureterorenoscopy and urinary drainage was preserved. This correlation of cross-sectional imaging with the ureteroscopic and intraoperative findings explains the preservation of renal architecture and the absence of upstream dilatation despite parenchymal and calyceal transit.

### 2.5 Three-dimensional reconstruction

Volumetric 3D CT reconstruction (Figures 1E,F) delineated the fragment trajectory relative to the renal hilum and major vessels, supporting the absence of major vascular encasement and defining the required dissection planes.

### 2.6 Ureterorenoscopy

Flexible ureterorenoscopy (Figure 1D) confirmed intrapelvic extension of the fragment and provided direct optical confirmation that the collecting system was not obstructed.

### 2.7 Rationale for excluding endoscopic-only retrieval

Given the transparietal course of the fragment through renal parenchyma into the calyceal system, and its size and planar configuration, endoscopic-only retrieval was judged unfeasible and was not attempted; an approach permitting circumferential dissection and controlled extraction was considered necessary to avoid fracture and parenchymal or vascular injury.

### 2.8 Rationale for laparoscopic transperitoneal surgery, and surgical technique

A laparoscopic transperitoneal approach was selected because it offered magnified visualisation adjacent to the renal hilum, a well-defined trajectory on 3D imaging, and the ability to complete circumferential dissection under direct vision. On 21 November 2025, elective laparoscopic transperitoneal removal was performed under general anaesthesia in the left lateral decubitus position. Pneumoperitoneum was established via a Veress needle (12 mmHg). An 11-mm umbilical camera port, a 5-mm right upper quadrant retraction port, and a 12-mm right iliac fossa working port were placed. The right colon was mobilised medially along the white line of Toldt, and the anterior layer of Gerota's fascia was incised.

**Figure 2:**
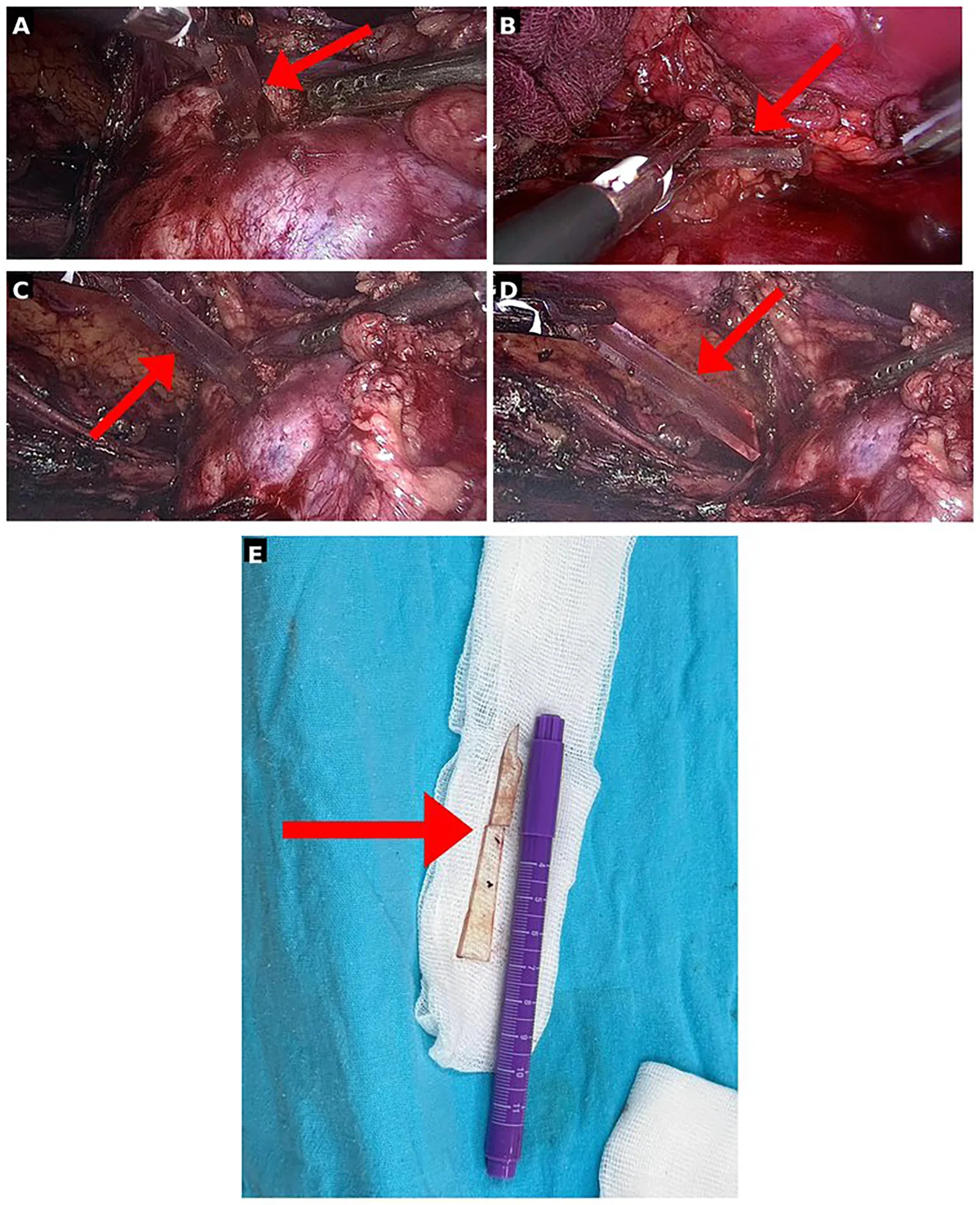
Intraoperative findings and retrieved specimen. **(A–D)** Sequential laparoscopic views during transperitoneal retroperitoneal dissection: progressive exposure of the glass foreign body (arrow) after medial mobilisation of the ascending colon and incision of Gerota's fascia, showing the foreign body traversing the quadratus lumborum and penetrating the renal capsule. The translucent planar morphology is visible under magnified laparoscopic illumination. **(E)** *Ex-vivo* photograph of the retrieved glass foreign body, recovered as two fragments (approximately 3 cm and 4 cm) alongside a calibrated ruler, corresponding to the two hyperdense structures identified on preoperative CT.

The foreign body was identified as translucent glass traversing the quadratus lumborum and penetrating the upper renal capsule (Figures 2A–D). Only minimal peri-fragment reactive fibrosis was encountered—an unexpected finding given the 25-year retention interval—which permitted direct laparoscopic localisation without fluoroscopy or intraoperative ultrasound. Layer-by-layer dissection with an ultrasonic device exposed the foreign body, which lay in two fragments along the penetration track: a proximal fragment (∼3 cm) within the quadratus lumborum and a distal fragment (∼4 cm) embedded in the renal parenchyma. Each fragment was freed circumferentially while continuous visualisation of the segmental vessels and hilum was maintained, and complete circumferential dissection was completed before any traction was applied, to avoid further glass fracture. Both fragments (Figure 2E) were grasped and delivered in a specimen bag through the 12-mm port; no further foreign material was identified in the dissection bed. Haemostasis was secured with bipolar coagulation and a topical haemostatic agent at the capsular entry point. A perinephric drain was placed. Operative time was 95 min and estimated blood loss was under 50 mL.

Complete removal of both fragments was confirmed intraoperatively by direct laparoscopic inspection of the dissection bed and by the retrieved specimen, which comprised two glass fragments (∼3 cm and ∼4 cm) corresponding to the two hyperdense structures identified on preoperative CT (Figure 2E).

### 2.9 Postoperative course and follow-up

The perinephric drain was removed on postoperative day 2. The patient remained in hospital for 7 postoperative days and was discharged in good general condition, with no recurrent gross haematuria and no postoperative complaints. Because she resided permanently in another region, she did not return for long-term follow-up; consequently, follow-up urinalysis, postoperative serum creatinine and eGFR, and postoperative ultrasound and CT were not available.

In the absence of postoperative cross-sectional imaging, radiological confirmation of long-term durability and of the absence of any residual foreign material cannot be provided; both intraoperative fragments were, however, retrieved in full and matched the two structures seen on preoperative CT.

## 3 Discussion

### 3.1 Focused literature review and comparison with previous reports

To provide clinical context for the present case, we performed a focused narrative review of the published literature to identify reports describing delayed presentation of retained glass or other traumatic retroperitoneal and renal foreign bodies. PubMed was searched using combinations of terms including “glass foreign body”, “retroperitoneal foreign body”, “renal foreign body”, “penetrating trauma”, and “laparoscopy”. Reference lists of relevant publications were also screened to identify additional eligible reports. Reports describing delayed presentation of retained foreign bodies involving the retroperitoneum, kidney, or other deep anatomical compartments were included for qualitative comparison (Table 2). To our knowledge, and based on this review, no previously published case describes a retroperitoneal glass foreign body with renal parenchymal involvement after a comparable 25-year retention interval.

**Table 2:** Published reports of retained foreign bodies of traumatic or iatrogenic origin, with delayed presentation, involving the retroperitoneum, kidney, or other deep anatomical compartments.

Author, Year	Material	Location	Retention	Presentation	Approach	Outcome
Ozsarac et al. (9)	Glass	Soft tissue, back	12–25 yrs	Pain, swelling	Open excision	Success
Na et al. (8)	Glass	Maxillofacial	>40 yrs	Facial swelling	Open excision	Success
Luks et al. (1)	Glass splinter	Abdominal cavity	9 months	Abdominal pain	Surgical removal	Success
Rajput et al. (2)	Metallic FB	Retroperitoneum	13 yrs	Pelvic pain	Laparoscopy	Success
Limbachia & Gandhi (4)	Surgical blade	Retroperitoneum	Iatrogenic	Pelvic pain	Laparoscopy	Success
Okuno et al. (3)	Metallic FB	Abdominal wall	Remote	Swelling, pain	Laparoscopy	Success
Başıbüyük et al. (11)	NR	Retroperitoneum	NR	Flank pain	Single-port laparoscopy	Success
Bedaiwi et al. (15)	Needle-like FB	Renal cortex	Iatrogenic	Flank pain	Laparoscopy	Success
Present case, 2025	Fractured retained glass foreign body (two fragments)	Retroperitoneum → kidney	25 yrs	Haematuria, colic	Laparoscopy	Success

FB, foreign body; NR, not reported.

The previously reported cases in Table 2 illustrate that long-retained deep foreign bodies are usually metallic or iatrogenic and are most often located in soft tissue, the abdominal wall, or the pelvis, with retrieval by open or laparoscopic means. Reported analogous retention intervals include glass retained for more than 40 years in the maxillofacial region (8) and a metallic instrument retained for 13 years in the retroperitoneum (2). The present case differs from these reports in the combination of features rather than in any single one: the material was glass; the location was retroperitoneal with migration into the renal parenchyma and calyceal system; the retention interval was exceptionally long; and management was by transperitoneal laparoscopy. To our knowledge this particular combination has not previously been described, although the single-case nature of the report limits any broader inference.

The prolonged, clinically silent course is consistent with glass being generally chemically stable *in vivo*; the surrounding tissue response may nonetheless vary with anatomical location and associated injury, so that within a relatively enclosed fibroadipose compartment a fragment may remain quiescent for long periods (9). Progressive renal penetration is most plausibly explained by gradual migration possibly influenced by postural, muscular, or gravitational forces (9); the original entry trajectory could not be reconstructed, and this mechanism should be regarded as speculative.

The exertional pattern of the haematuria is clinically distinctive. A plausible mechanism is intermittent mechanical irritation or microtrauma of the renal parenchyma or calyceal mucosa during physical activity, producing transient bleeding; this remains a hypothesis and was not directly demonstrated.

### 3.2 Diagnostic strategy: CT, 3D reconstruction, and trauma history

The 12-month diagnostic delay reflected two potentially avoidable factors: the remote penetrating trauma was not initially elicited, and the glass density was not recognised on the first CT. Once the trauma history was available, the CT appearance was more readily interpretable. The fragments measured ∼1,150–1,200 HU; urinary calculi show variable attenuation depending on composition, with calcium-oxalate stones averaging roughly 600 HU and uric-acid stones lower (approximately 300–450 HU) (14), so that attenuation together with the linear, extracalyceal morphology and the remote trauma history favoured a glass foreign body over a calculus and clearly distinguished it from soft tissue.

Volumetric 3D reconstruction added information not available on axial imaging alone: simultaneous three-plane depiction of the oblique trajectory, assessment of the relationship to major vessels, and definition of dissection planes, which informed port placement. In this case, multiphase CT and 3D reconstruction were sufficient to exclude major vascular encasement; however, CT angiography may be warranted when vascular involvement remains uncertain. Ureterorenoscopy provided optical confirmation of intrapelvic extension. In our experience, a systematic trauma-history inquiry is a simple and valuable measure in any patient with unexplained haematuria in whom CT does not demonstrate a convincing calculus.

### 3.3 Laparoscopic surgical principles

Three considerations favoured a laparoscopic transperitoneal approach: a well-defined fragment trajectory on 3D imaging, the absence of a large haematoma or dense fibrosis, and the need for magnified dissection adjacent to major retroperitoneal vessels. Published reports generally support laparoscopy as the preferred approach in haemodynamically stable patients without extensive adhesions (2–4, 11, 15), and the present case is consistent with that experience for a long-retained glass foreign body. A technical point specific to glass is the avoidance of fracture: complete circumferential dissection before any traction, with delivery in a specimen bag, distributes extraction force along the long axis and reduces the risk of further fragmentation. The limited fibrous encapsulation encountered after 25 years facilitated dissection; whether this can be expected at comparable intervals in other cases is uncertain, and preoperative exclusion of vascular involvement remains essential.

### 3.4 Limitations

This report is limited by its single-case design, from which no generalisable conclusions can be drawn. The original entry trajectory could not be reconstructed, and the proposed migration mechanism is therefore speculative. Histopathological examination was not performed because no macroscopic evidence of abscess, fibrosis, or suspicious tissue change was identified intraoperatively; formal histology was therefore not considered clinically indicated. Because the patient resided in another region and did not return for review, postoperative follow-up was limited to the inpatient period, and follow-up urinalysis, serum creatinine and eGFR, and postoperative ultrasound or CT were not obtained; long-term durability of the outcome therefore cannot be established radiologically, although both foreign-body fragments were retrieved in full and corresponded to the two structures identified on preoperative CT.

## 4 Conclusion

A glass foreign body retained for 25 years in the retroperitoneum, recovered as two fragments with apparent progressive renal parenchymal penetration, represents—to our knowledge, and based on the available published literature—an unusually long latency for this clinical scenario. Within the limits of a single case, three practical points emerge: a systematic trauma history is valuable in unexplained gross haematuria; multiphase CT with 3D reconstruction can serve both diagnostic and surgical-planning roles; and laparoscopic transperitoneal removal, with complete pre-extraction dissection, was safe and effective in this patient once preoperative imaging had excluded vascular encasement. Long-term radiological follow-up would be desirable to confirm the durability of the outcome; such data were, however, unavailable in the present case.

## Data Availability

The original contributions presented in the study are included in the article. Further inquiries can be directed to the corresponding author.
